# Edible Bird's Nest Prevents Menopause-Related Memory and Cognitive Decline in Rats via Increased Hippocampal Sirtuin-1 Expression

**DOI:** 10.1155/2017/7205082

**Published:** 2017-09-20

**Authors:** Zhiping Hou, Peiyuan He, Mustapha Umar Imam, Jiemen Qi, Shiying Tang, Chengjun Song, Maznah Ismail

**Affiliations:** ^1^Department of Pathology, Chengde Medical University, Chengde, Hebei 067000, China; ^2^Laboratory of Molecular Biomedicine, Institute of Bioscience, Universiti Putra Malaysia, 43400 Serdang, Selangor, Malaysia; ^3^Gastroenterology Department, Affiliated Hospital of Chengde Medical University, Chengde, Hebei, China; ^4^Precision Nutrition Innovation Center, School of Public Health, Zhengzhou University, Zhengzhou 450001, China

## Abstract

Menopause causes cognitive and memory dysfunction due to impaired neuronal plasticity in the hippocampus. Sirtuin-1 (SIRT1) downregulation in the hippocampus is implicated in the underlying molecular mechanism. Edible bird's nest (EBN) is traditionally used to improve general wellbeing, and in this study, we evaluated its effects on SIRT1 expression in the hippocampus and implications on ovariectomy-induced memory and cognitive decline in rats. Ovariectomized female Sprague-Dawley rats were fed with normal pellet alone or normal pellet + EBN (6, 3, or 1.5%), compared with estrogen therapy (0.2 mg/kg/day). After 12 weeks of intervention, Morris water maze (four-day trial and one probe trial) was conducted, and serum estrogen levels, toxicity markers (alanine transaminase, alkaline phosphatase, urea, and creatinine), and hippocampal SIRT1 immunohistochemistry were estimated after sacrifice. The results indicated that EBN and estrogen enhanced spatial learning and memory and increased serum estrogen and hippocampal SIRT1 expression. In addition, the EBN groups did not show as much toxicity to the liver as the estrogen group. The data suggested that EBN treatment for 12 weeks could improve cognition and memory in ovariectomized female rats and may be an effective alternative to estrogen therapy for menopause-induced aging-related memory loss.

## 1. Introduction

Estrogen regulates the development and functioning of the central nervous system [1, 2]. Decreased serum estrogen levels after menopause or ovariectomy have been shown to promote inflammatory pathology involving oxidative stress [3] and can be a risk factor for neurodegenerative diseases such as Alzheimer's disease. Recent studies have suggested the preventive effects of hormone replacement therapy (HRT) or phytoestrogen supplementation therapy on oxidative stress-mediated neurodegenerative disorders [4]. However, it has been demonstrated that HRT in postmenopausal women can lead to the development of breast, ovarian, and endometrial cancers [5–7]. Thus, an alternative phytoestrogen treatment might be of benefit compared with conventional HRT that has adverse effects.

Sirtuin-1 (SIRT1) is a member of the sirtuin family, which is known to regulate intracellular regulatory proteins with mono-ADP-ribosyl transferase activity. SIRT1 is reported to improve insulin sensitivity and affect the essential metabolic regulatory transcription factors including those of the PGC1-alpha/ERR-alpha complex [8]. Similarly, SIRT1 was demonstrated to regulate energy metabolism, stress resistance, neurodegeneration, and senescence [9–11].

Edible bird's nest (EBN) originates from the saliva of swiftlet species; they mostly come from *Collocalia fuciphaga*, *Aerodramus fuciphagus*, and *Aerodramus maximus* species, commonly found in Southeast Asia [12, 13]. EBN has been considered a precious food tonic by Chinese people ever since the Tang dynasty (618 AD) [14], and its usage in present times is principally based on historical and observational results of its beneficial effects including antiaging and immune-enhancing properties [15]. More recent scientific evidence suggests that EBN is both nutritionally and functionally rich [16–18]. Its components include lactoferrin, sialic acid, ovotransferrin, minerals, and amino acids including essential amino acids, such as lysine, tyrosine, and serine [16, 17, 19]. There is some evidence to support its anti-inflammatory, antioxidant, and insulin-sensitizing effects [19–22]. It could also improve and attenuate age-related neurodegenerative changes [23]. However, there is no evidence suggesting that EBN could improve memory and cognition as part of its overall anti-aging properties despite its long history of medicinal use.

Thus, the present study was designed to investigate the effects of EBN on ovariectomy-induced cognitive dysfunction, especially in relation to changes in SIRT1 function.

## 2. Materials and Methods

### 2.1. Animal Treatment and Operation Procedure

Forty-two Sprague-Dawley rats (3 months old, female, 180–200 g) were housed under controlled conditions (12 h light/12 h dark cycle, 20–22°C, 40–50% humidity) with access to water and food ad libitum for two weeks prior to the experiments for acclimatization to the new environment. The use of animals was approved by the Animal Care and Use Committee (ACUC) of the Faculty of Medicine and Health Sciences, Universiti Putra Malaysia (Project approval number: UPM/IACUC/AUP-R012/2014), and animals were handled as stipulated by the guidelines for the use of animals. All ovariectomy (OVX) procedures were done as previously described [24] and were conducted under anesthesia with an injection of 10 mg/60 mg/kg xylazine/ketamine (i.p.). Briefly, bilateral OVX was performed from a dorsal approach after shaving the fur on both sides of the body, after which, the ovaries and the surrounding tissue were removed; the incisal opening was closed by stapling the muscles and suturing the skin [24]. The control group was not operated on, while the sham group underwent a sham surgery, in which, only skin and muscles were cut but the ovaries were spared. After OVX, the rats were maintained for one week and randomly assigned to seven groups (*n* = 6): OVX group, ovariectomized and received daily standard rat chow; OVX + estrogen group, ovariectomized and received 0.2 mg/kg body weight/day of estrogen orally in addition to standard rat chow; and OVX + EBN high dose, OVX + EBN medium dose, and OVX + EBN low dose groups, ovariectomized and received 1.2, 0.6, and 0.3 g/kg body weight/day of EBN, respectively, in addition to standard rat chow. Treatments lasted for 12 weeks, during which, food intake was measured daily and weight was measured weekly.

### 2.2. Morris Water Maze (MWM) Behavioral Test

The MWM apparatus consisted of a black circular plastic pool that measured 170 cm in diameter and 60 cm in height and a cylindrical dark colored platform with a diameter of 10 cm. It was placed in a light-controlled room, and curtains with three black distal cues were mounted around the maze. The water temperature in the pool was maintained at 22 ± 1°C, and recording was done using the ANY-maze Video Tracking System (Stoelting, Wood Dale, IL, USA), connected to a CCD camera and used to assess performance and reference memory in the water maze task. The platform was placed in the middle of the target quadrant and submerged 2 cm below water's surface and kept at the same position throughout the experiment.

The spatial acquisition phase consisted of four training days and four training trials per day per rat. The start position used distal locations for which the hidden platform is located in the northwest quadrant during the acquisition phase. The sequences of starts were designed such that the platform was to the right or left of an animal during an equal number of trials, and one trial was performed from each of the four start positions each day. Rats were released facing the pool wall at water level from the desired start position (E, S, SW, and NE tactic) and allowed to search for the platform for 60 s. If rats did not find the platform within the limited time, animals were guided to the platform and left there for 15 s. The rats were then placed in the maze after a short rest at a new start location, and the latter three trials were repeated. This was done for a total of four days. The spatial memory was evaluated by the latency (time from start to platform) and path length.

Reference memory (probe trial) version was done on the fifth day without a platform, allowing each rat to swim freely for 30 s. The rats were placed at the southeast position (opposite the target quadrant in the spatial acquisition phase). The reference memory was evaluated by the time the rat stayed in the target quadrant.

### 2.3. Preparation of Tissue Samples

At the end of the experiment, all animals were decapitated and exsanguinated after anesthesia with an injection of 10 mg/60 mg/kg xylazine/ketamine (i.p.). The hippocampus was removed from the brain and quickly kept in the RCL2 reagent (Alpjelys, Toulouse, France) for further analysis of molecular markers.

### 2.4. Serum Biochemical Analysis

#### 2.4.1. EBN Toxicity

Blood collected after sacrifice was centrifuged at 3000*g* for 15 mins at 4°C. The supernatant was collected and stored at −80°C. Liver enzymes (ALT, ALP, and GGT) and kidney function markers (urea and creatinine) were determined on the Dimension Xpand Plus Integrated Chemistry System (Siemens, Germany) with commercially available kits (Randox Laboratories Ltd., Antrim, UK).

#### 2.4.2. Serum Estrogen Detection

Serum estrogen levels were determined by the commercial ELISA kit (Cusabio, Wuhan, China) in accordance with the manufacturer's instruction.

### 2.5. Sirtuin-1 Immunohistochemistry

For SIRT1 immunohistochemistry, paraffin sections (3 *μ*m) were placed in an oven at 60°C for 30 min. The sections were then deparaffinized and rehydrated by xylene twice and gradient ethanol from pure to 70%. Heat-induced retrieval of antigen was done by 10 mM sodium citrate (pH 6.0), 3% hydrogen peroxide for 10 mins to suppress the endogenous peroxidase activity, and 1% bovine serum albumin (all from Sigma, St. Louis, MO, USA) for 15 mins to block nonspecific binding. After this, the sections were incubated in rabbit polyclonal anti-SIRT1 (Abnova-PAB0004, Abnova Corporation, Taipei, Taiwan) antiserum at a dilution of 1 : 225 overnight at 4°C. After washing in TBS-T, the sections were incubated with a HRP-conjugated secondary antibody (1 : 400) at room temperature for 1 hr, followed by the DAB staining for 10 min at room temperature. Water was then used to rinse for 5 min followed by counterstaining with hematoxylin. Finally, the sections were dehydrated and mounted to evaluate SIRT1 immunohistochemistry in the hippocampus. All the sections were captured by the confocal laser scanning microscope (LSM 5 Pa) equipped with the acquisition software ZEN 2007 (both from Carl Zeiss MicroImaging GmbH, Jena, Germany). Hippocampal neurons with 10 random circles in each image were recorded, and their densitometric readings were combined together and averaged to get the total optical density (TOD) [25]. In order to avoid bias, the background staining (BOD) of each image was measured as TOD. The expected OD of each image was the gap between TOD and BOD. For the minus control of immunohistochemistry, PBS instead of primary antibody was used (data not shown).

### 2.6. Statistics

All statistical analyses were done using SPSS software (version 20.0). The parameters that were evaluated in the MWM special acquisition and reference memory were analyzed by repeated measure analysis of variance and one-way or two-way ANOVA, respectively. Statistical data were expressed as means ± SEM. The criteria for statistical significance was defined as *P* < 0.05.

## 3. Results

### 3.1. Food Intake, Body Weight, and Estrogen Levels

Body weights were similar at the beginning of the experiment, but at the end of the intervention, the OVX group had significantly higher body weight than the sham group (Table 1). After 12 weeks of EBN supplementation, the OVX + EBN groups had significantly lower body weights in comparison with the OVX group. Similarly, after 12 weeks of treatment, the OVX group had higher food intake compared to the sham group, while the group with EBN and estrogen supplementation significantly had reduced food intake compared to the OVX group. Specifically, the OVX + EBN high-dose group had similar food intake to the OVX + estrogen group. Serum estradiol levels were found to be significantly lower in the OVX group and higher in the EBN groups compared with the sham group. The reduced serum estradiol levels and increased food intake and weight gain in the OVX group suggested that the OVX model was successful, since EBN could regulate food intake, weight gain, and estrogen levels especially at higher doses.

### 3.2. Morris Water Maze

**Figure 1:**
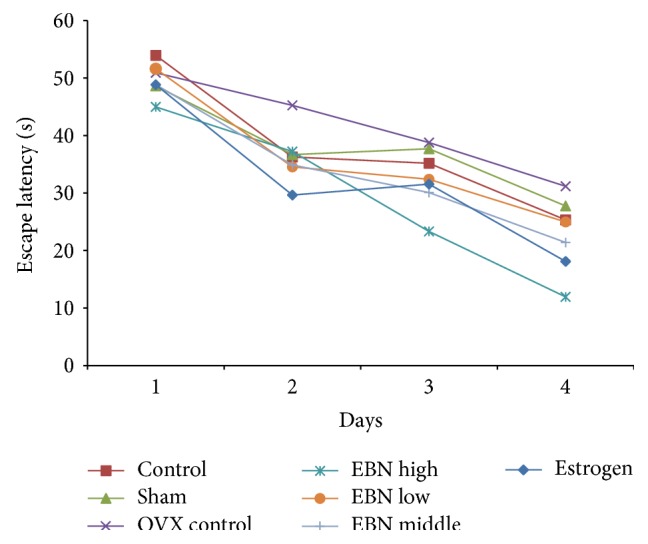
Effect of EBN on MWM performance in the spatial memory acquisition phase. All values are expressed as mean ± SEM for 6 animals in each group. Sham: surgically opened but not ovariectomized and fed with standard rat chow for 12 weeks. OVX: ovariectomized and fed with standard rat chow for 12 weeks; estrogen: ovariectomized and fed with standard rat chow and 0.2 mg/kg body weight of estrogen for 12 weeks; EBN high, medium, and low: ovariectomized and fed with standard rat chow and 1.2, 0.6, and 0.3 g/kg body weight of EBN/day, respectively, for 12 weeks.

To determine the dose efficiency of EBN supplementation on learning and memory in ovariectomized rats, behavioral performances were compared among all the groups which received the MWM test before sacrifice.  Figure 1 shows that rats from all groups learned the task well and displayed a gradual decrease in escape latencies over the 4-day training acquisition (F (3, 140) = 352.1; *P* < 0.001). The control and sham groups had no changes in escape latencies (F (1, 40) = 6.575*e*−005; *P* = 0.9936). Furthermore, the rats in the EBN high-dose group were faster than those in the estrogen group on escape latencies. The MWM revealed that the escape latency (the average time to find the hidden platform) in EBN treatment groups was considerably decreased from low dose to high dose.

**Figure 2:**
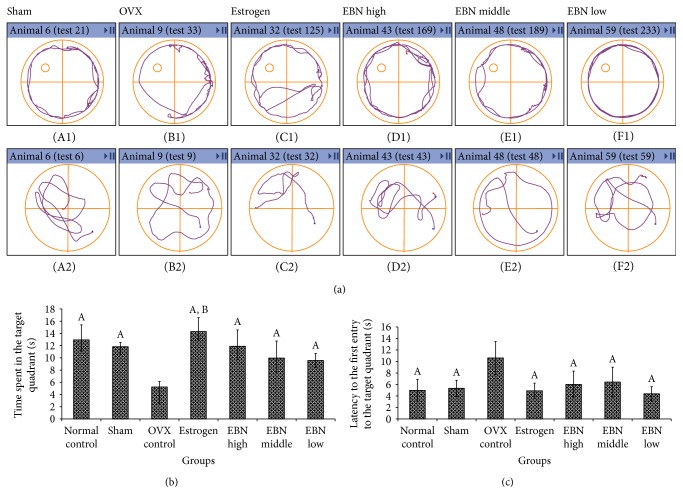
(a) Representative path tracings of the Morris water maze. A: sham group (A1, first day first trial; A2, probe trial); B: OVX group (B1, first day first trial; B2, probe trial); C: estrogen group (C1, first day first trial; C2, probe trial); D: EBNH group (D1, first day first trial; D2, probe trial); E: EBNM group (E1, first day first trial; E2, probe trial); F: EBNL group (F1, first day first trial; F2, probe trial). (b) Time in the target quadrant in the probe trial; (c) latency to the first entry to the target quadrant. ^A^*P* < 0.05 compared with OVX group; ^B^*P* < 0.05 compared with sham group for spending time in the target quadrant and latency to the first entry to the target quadrant (ANOVA). OVX: ovariectomized. Groups are the same as in Figure 1.

Figure 2(a) shows the representative path tracings of the MWM. There was general preference for the target quadrant in the spatial version (lower panel) and the preference of circling for the same animals in the spatial version (upper panel). Two-way ANOVA revealed that the OVX group showed lower ability to learn and find the target platform compared to the sham group (F (1, 40) = 12.01; *P* = 0.0013). In contrast, the group with estrogen and EBN supplementation (0.6 and 1.2 g/kg/day) showed strong ability to find the target platform compared to the sham group (F (3, 80) = 24.59; *P* < 0.0001).

In the probe test, swimming time (min) in the target quadrant for each group was as follows: estrogen (14.63 ± 2.23) > EBN high dose (12.73 ± 2.02) > control (12.5 ± 2.19) > EBN middle dose (10.58 ± 2.37) > sham (10.50 ± 0.62) > EBN low dose (10.23 ± 2.47) > OVX (4.65 ± 0.81). There were no significant differences for the time in the target quadrant (Figure 2(b)) between the control and sham groups (*P* > 0.05). However, rats in the OVX group spent less time in the target quadrant compared with those in the sham group (*P* < 0.01). Similarly, the estrogen and EBN groups' data showed more time spending on the target quadrant compared to those of the OVX group. The latency to the first entry to the target quadrant (Figure2(c)(c)) of each group was determined as follows: estrogen (4.90 ± 1.33) < control (4.97 ± 1.93) < EBNL (4.38 ± 1.22) < sham (5.33 ± 1.40) < EBNH (6.02 ± 2.33) < EBNM (6.42 ± 2.59) < OVX (10.62 ± 2.87).

### 3.3. Toxicity Evaluation

**Figure 3:**
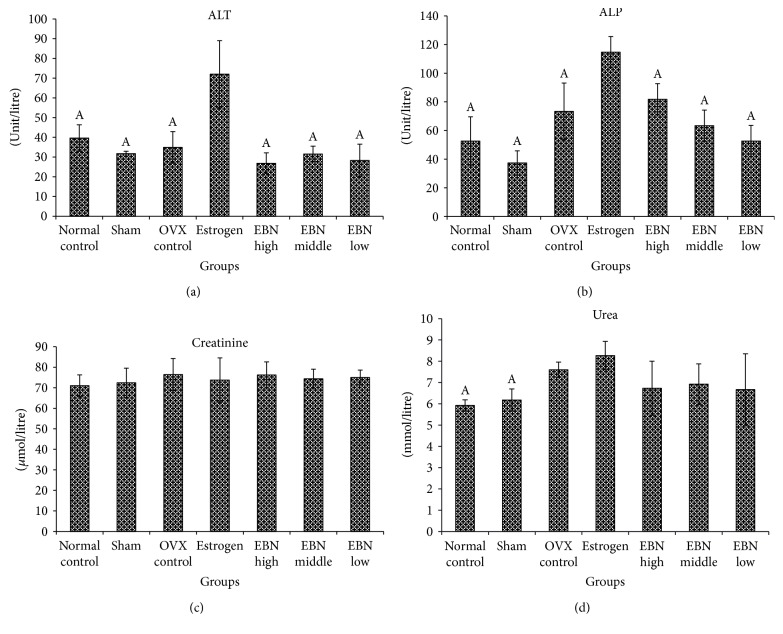
Liver enzymes and kidney function. (a) Serum ALT activity; (b) serum ALP activity; (c) serum creatinine activity; (d) serum urea activity. Data are expressed as mean ± SEM of six animals. ^A^*P* < 0.01 compared with estrogen group. Groups are the same as in Figure 1.

Rats treated (p.o.) with estrogen demonstrated increases in ALT and ALP enzyme activities by 128% and 207%, respectively, compared to the sham group (Figures 3(a) and 3(b)). Conversely, the EBN treatment did not increase the levels of these enzymes as much as estrogen did. The serum creatinine levels (Figure 3(c)) were similar for all groups, while serum urea (Figure 3(d)) was marginally elevated in the treatment groups compared to the control and sham groups.

### 3.4. SIRT1 Expression in the Hippocampus

**Figure 4:**
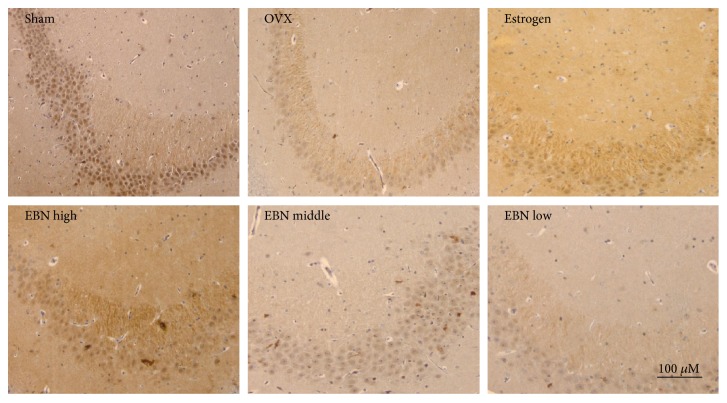
SIRT1 immunoreactivity in the pyramidal layer of the hippocampus (100x). Representative micrographs of SIRT1 (*n* = 6) show the pyramidal layer of the hippocampus. The age-associated reduction in SIRT1 immunoreactivity is attenuated by OVX and ameliorated by EBN treatment. Groups are the same as in Figure 1.

**Figure 5:**
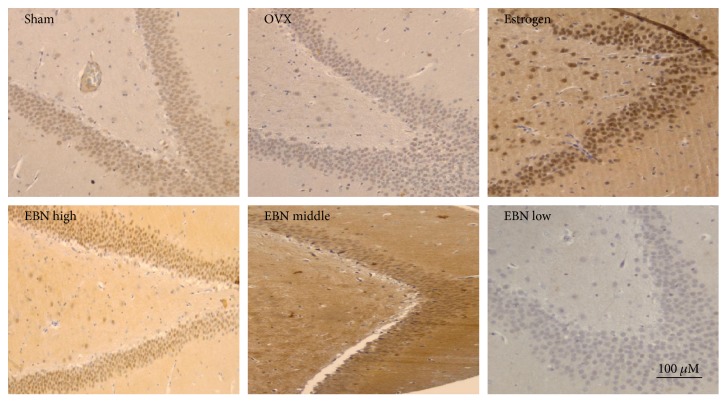
SIRT1 immunoreactivity in the dentate gyrus (100x). Representative micrographs of SIRT1 (*n* = 6) in the dentate gyrus demonstrate that the age-associated reduction in SIRT1 immunoreactivity is attenuated by OVX and ameliorated by EBN treatment. Groups are the same as in Figure 1.

**Figure 6:**
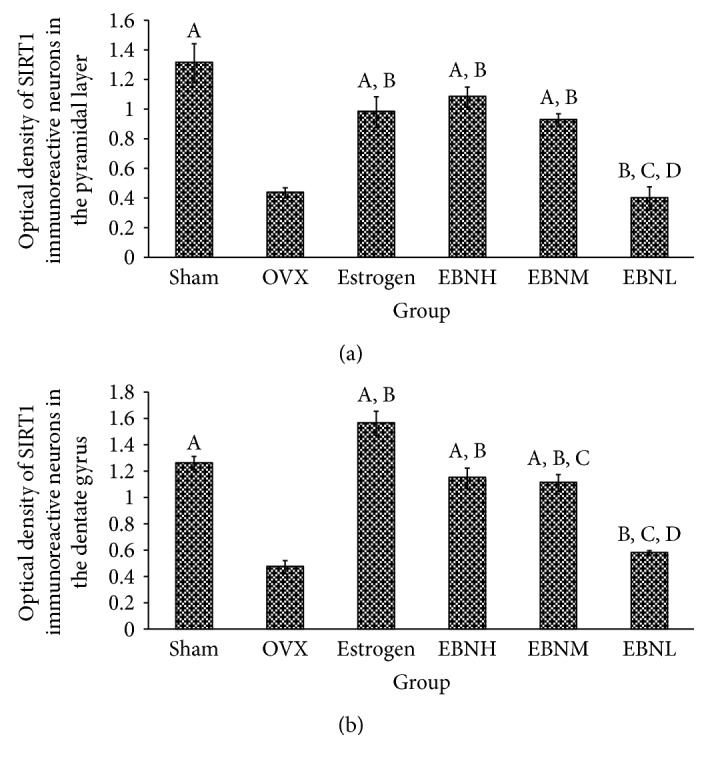
Bar graphs showing the optical density (OD) of SIRT1 immunoreactivity in the pyramidal layer (a) and dentate gyrus (b). ^A^*P* < 0.05 compared with OVX; ^B^*P* < 0.05 compared with sham operation group; ^C^*P* < 0.05 compared with estrogen; ^D^*P* < 0.05 compared with EBN group. Groups are the same as in Figure 1.

The SIRT1-immunoreactive neurons were observed and analyzed in the pyramidal layer of the hippocampus (Figure 4) and the dentate gyrus (Figure 5). In the sham-operated group, numerous neurons in the hippocampal formation were darkly stained with SIRT1 immunohistochemistry. However, for the OVX group, the SIRT1 immunoreactivity was drastically decreased. Similarly, the immunoreactivity in the estrogen and EBN groups was moderate to strong in the dentate gyrus and pyramidal neuron in the CA2 area. Quantitative analysis (Figure 6) revealed that the optical density of hippocampal SIRT1 staining was significantly reduced from 1.31 ± 0.13 (pyramidal neuron) and 1.25 ± 0.05 (dentate gyrus) in the sham-operated group to 0.44 ± 0.03 (pyramidal neuron) and 0.46 ± 0.09 (dentate gyrus) in OVX rats (Figure 4).

## 4. Discussion

The present study provides evidence that cognitive function could be affected by EBN treatment through improving SIRT1 expression in the hippocampus of ovariectomized female rats. We observed that the preservative effect of EBN was dose-dependent on cognition and hippocampal neuronal SIRT1. Moreover, EBN treatment was less toxic compared to estrogen therapy, which is commonly used in HRT for menopausal symptoms.

The MWM is primarily a test of spatial learning and reference memory [26, 27]. During the navigation test, there was a decrease in escape latencies across successive four days in all the groups. This indicated that under normal physiological or neurodegenerative conditions, four trainings per day can establish the effective reference memory, subsequently improving the next day's navigation performance [27]. However, at the fourth day training, the OVX group had the longest escape latency compared with the sham and EBN treatment groups, suggesting that the spatial learning ability was impaired in these rats. The effect of EBN treatment was very similar to that of the estrogen treatment suggesting that EBN was as effective as estrogen in improving menopause-related cognitive decline.

Spatial learning and memory in rodents are mainly associated with hippocampal function and morphology [28]. Thus, the present findings suggest that the ability of EBN to improve memory and learning in ovariectomized rats may be tied to its effects on SIRT1 expression in the pyramidal layer and dentate gyrus of the hippocampus. SIRT1 is an enzyme that may increase lifespan through mediation of neuronal plasticity [29–31]. Furthermore, estrogen is able to enhance hippocampal SIRT1 expression as the basis for its ability to improve neuronal plasticity critical for modulating learning and memory [2, 3]. However, because estrogen is associated with adverse effects [5–7], the search for safer alternatives is ongoing. Moreover, the toxicity results in the present study showed that ALT, ALP, and urea in the estrogen group were significantly higher than those in other groups. Taken together, the present toxicity data corroborates the adverse effects of estrogen supplementation. Therefore, although estrogen can improve learning and memory deficits of OVX rats, its effects on liver enzymes and kidney function are unwanted.

Furthermore, EBN is known to possess multiple bioactive compounds that synergistically contribute to its bioactivity [16–18]. Moreover, we have demonstrated previously that the two major components of EBN (lactoferrin and ovotransferrin) could not account for the entire antioxidant effects of EBN [19] suggesting that the presence of other compounds may enhance the overall effects of EBN. This is in line with the concepts of food synergy [32] and bioactive rich fraction [33], which propose that interactions between multiple food nutrients or plant phytochemicals and their relationship with the food matrix may play a more significant role in producing the beneficial effects of foods or plant phytochemicals over any single nutrient present in the food or plant.

## 5. Conclusions

This study has shown for the first time that EBN treatment preserved hippocampal SIRT1 activity in adult female rats subjected to OVX-related aging, which may have been the basis for the cognitive-enhancing properties of EBN. The positive function of EBN is probably mediated by enhancing the SIRT1-mediated neuronal plasticity that contributes to normal cognitive activity. Although the detailed cellular and molecular mechanisms related to OVX-induced cognitive impairment still remain unclear, the potent neuroprotective effects of EBN suggest that EBN could be an attractive candidate and novel strategy for the management of cognitive dysfunction associated with menopause.

## Figures and Tables

**Table 1:** Body weights and serum estrogen levels of ovariectomized rats after 12 weeks.

	Body weight (g) before treatment	Body weight (g) end point	Total weight gain (g)	Total food intake (g)	Serum estrogen (pg/ml)
Control	221.5 ± 33.5	227.5 ± 6.0	6.0	1109.3 ± 40.6	151.1 ± 8
Sham	217.8 ± 44.0	224.6 ± 6.8	6.8	1065.4 ± 23.8	156.7 ± 13
OVX	230 ± 29.7	283.1 ± 23.1^b^	53.1^b^	1276.2 ± 56.9^b^	35.6 ± 0.9^b^
Estrogen	236 ± 33.3	239 ± 3.0^a^	3.0^a^	976.8 ± 47.5^a,b^	169.8 ± 11.4^a^
EBN (high)	213.1 ± 41.8	214.5 ± 1.4^a^	1.4^a^	1023.5 ± 23.3^a^	150.4 ± 7.4^a^
EBN (middle)	228.1 ± 53.9	242.8 ± 14.6^a^	14.6^a^	1088.6 ± 40.4^a^	147.8 ± 8.7^a^
EBN (low)	216 ± 31.5	242.6 ± 26.7	26.7^a^	1111.5 ± 37.6^a^	143.3 ± 13.4^a^

Control: standard rat chow; sham: surgically opened but not ovariectomized and fed with standard rat chow; OVX: ovariectomized with semipurified pellet; estrogen: ovariectomized and fed with standard rat chow and 0.2 mg/kg body weight of estrogen; EBN high, medium, and low: ovariectomized and fed with standard rat chow and 1.2, 0.6, and 0.3 g/kg body weight of edible bird's nest/day, respectively. Values are mean ± SD, *n* = 5 − 6. ^a^Mean value was significantly different from that of the OVX group (*P* < 0.05); ^b^mean value was significantly different from that of the sham group (*P* < 0.05).

## Abbreviations

EBN: Edible bird's nest
OVX: Ovariectomy
MWM: Morris water maze
SIRT1: Sirtuin-1
ALP: Alanine transaminase
ALT: Alkaline phosphatase.
